# Extrachromosomal DNA and cancer: function, formation, and clinical implications

**DOI:** 10.1186/s13046-026-03639-0

**Published:** 2026-01-19

**Authors:** Yucai Wu, Rui Rui, Tai Tian, Jiaying Zheng, Shiming He, Liqun Zhou, Xuesong Li, Yanqing Gong

**Affiliations:** 1 Department of Urology, Peking University First Hospital, Beijing, 100034 China; 2 Institute of Urology, Peking University, Beijing, 100034 China; 3 National Urological Cancer Center, Beijing, 100034 China; 4 Urogenital Diseases (Male) Molecular Diagnosis and Treatment Center, Peking University, Beijing, 100034 China; 5 Sun Yat-Sen University, Guangzhou, 510080 Guangdong Province China

**Keywords:** Extrachromosomal DNA, Extrachromosomal circular DNA, Tumor heterogeneity, Therapeutic resistance, Gene amplification

## Abstract

Extrachromosomal DNA (ecDNA) is a circular, double-stranded DNA molecule distinct from chromosomal DNA, primarily found in cancer cells as a subtype of extrachromosomal circular DNA (eccDNA). Unlike eccDNAs found in normal cells, cancer-associated ecDNAs are large, clonal, and carry complete oncogenes. These ecDNAs are increasingly recognized as crucial drivers of cancer pathogenesis, contributing significantly to the evolution of tumor heterogeneity and the acquisition of therapeutic resistance through mechanisms such as high-level gene amplification and altered gene regulation. Historically, the understanding of these mobile genetic elements in cancer has been limited. This review synthesizes current knowledge on ecDNA’s structural and functional features, formation mechanisms and roles in cancer initiation, progression and therapeutic resistance. Moreover, we summarize five emerging therapeutic approaches that target ecDNA to inform future cancer research and precision medicine.

**Graphical Abstract:**

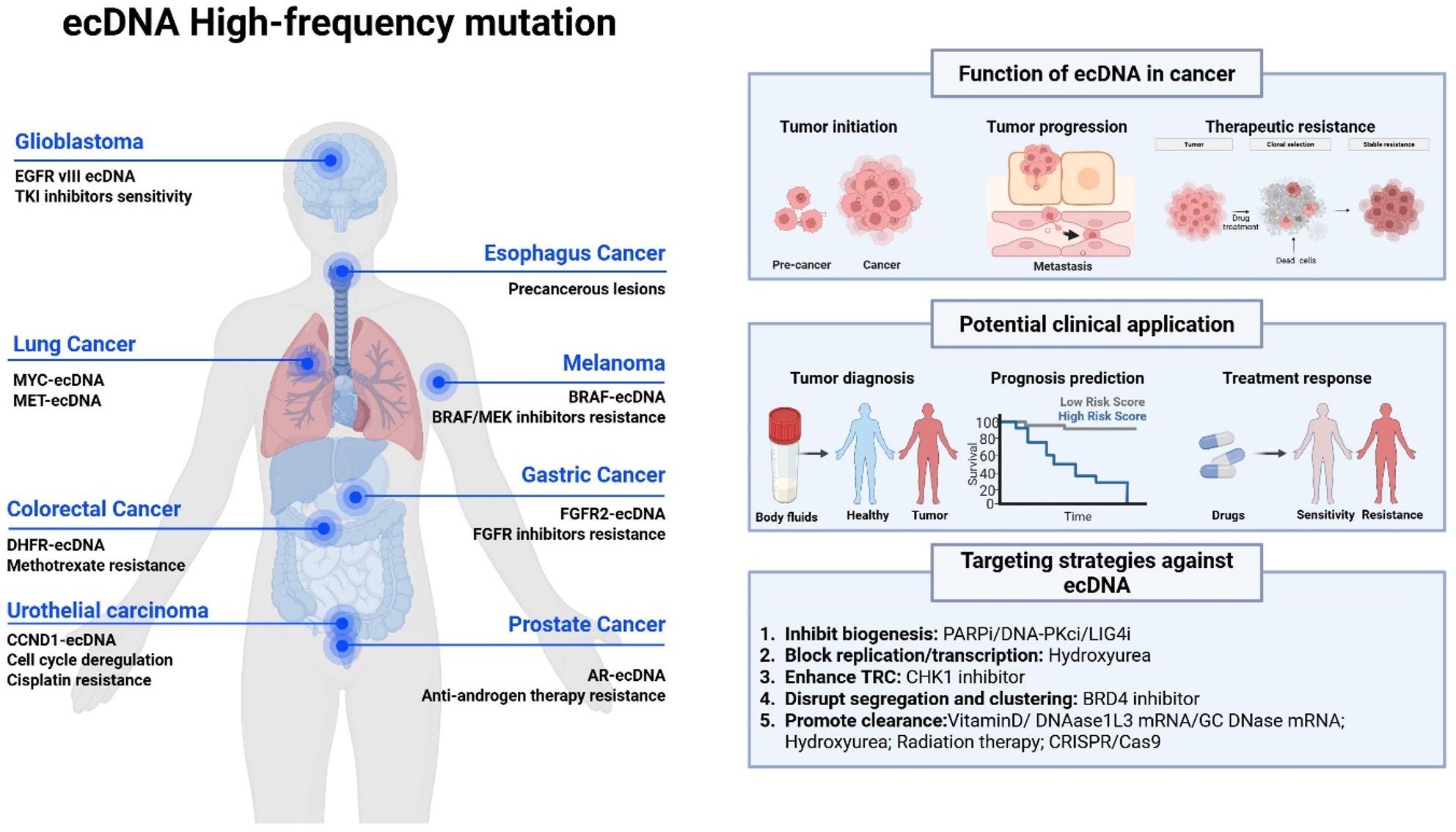

## Background

The development and progression of cancer are commonly believed to be driven by structural alterations in chromosomal DNA, including gene mutations, rearrangements, and amplifications [1]. However, recent advances have highlighted extrachromosomal DNA (ecDNA) as a previously underestimated yet functionally critical component of cancer genome complexity. EcDNA refers to circular DNA molecules located in the nucleus but independent of chromosomes, typically ranging in size from 100 kilobases (kb) to several megabases (Mb) [2]. Discovered in the 1960s, its biological role remained obscure for decades due to technical constraints [3]. These structures often appear in pairs under the microscope and were therefore historically termed double minutes (DMs) [4].

With the advent of single-cell sequencing, structural variant detection algorithms, and 3D genome technologies, ecDNA has been increasingly recognized as a pervasive feature in malignancies such as lung cancer, glioblastoma, and neuroblastoma [5–7]. Unlike chromosomal DNA, ecDNA lacks a centromere, which prevents it from being equally segregated into daughter cells during mitosis [8]. As a result, ecDNA exhibits asymmetric segregation during somatic cell mitosis, contributing to pronounced genetic heterogeneity among tumor cells [8]. EcDNA frequently carries oncogenes [9], drug resistance genes [10], and immune regulatory genes [11], which collectively underpin tumor progression, poor prognosis, therapy resistance, and immune evasion. In addition, ecDNA may also harbor enhancer elements that form long-range enhancer hijacking networks to aberrantly activate oncogene expression [12]. The formation of ecDNA is closely linked to deficiencies in DNA repair mechanisms, particularly nonhomologous end joining (NHEJ) and microhomology-mediated end joining (MMEJ) [13]. These error-prone pathways result in ecDNA production as a direct consequence of genomic instability and further promote the evolutionary adaptability of tumor cells [13, 14]. It is worth noting that ecDNA is distinct from extrachromosomal circular DNA (eccDNA), which is typically smaller (100 base pairs to several kb) and is found in both cancer and normal cells [2, 15].

In this review, we dissect the current understanding of ecDNA’s structural and functional features, formation mechanisms and its roles in cancer initiation, progression and therapeutic resistance. Furthermore, we summarize the current therapeutic strategies targeting ecDNA and emphasized its potential for clinical translation.

## The structural and functional characteristics of EcDNA

### EcDNA is a circular double-stranded molecule

**Fig. 1:**
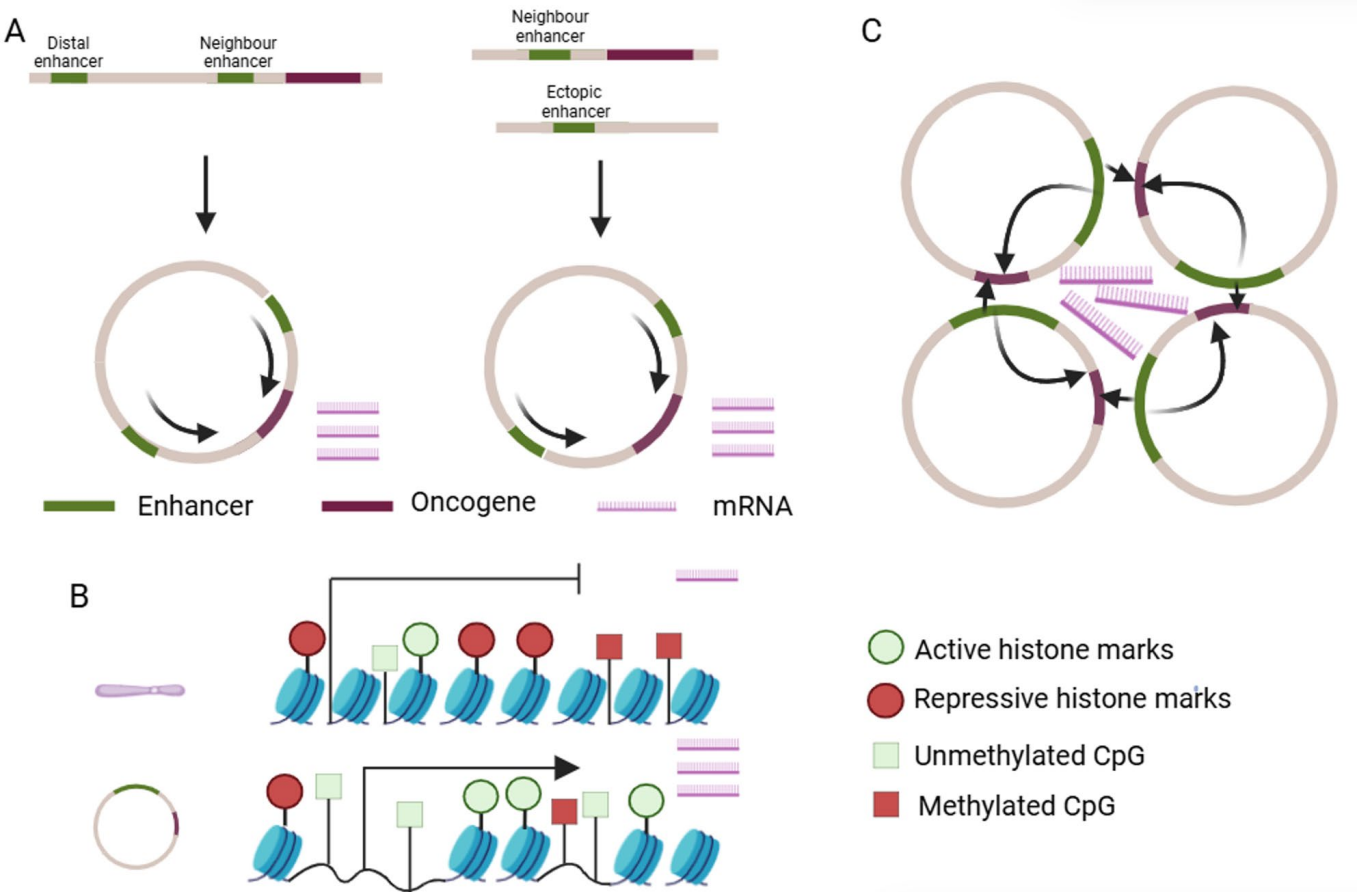
The structural and functional characteristics of ecDNA. **A** Enhancer hijacking: ecDNA can hijack enhancers located at distal regions of the same chromosome (left panel) or from different chromosomes (right panel). **B** Compared with chromosomal DNA, ecDNA exhibits a more open chromatin state, characterized by increased active histone modifications, reduced repressive histone marks, and lower promoter methylation levels. **C** EcDNAs form hubs to enhance transcription. Figure created using BioRender (biorender.com)

EcDNA is a circular, double-stranded DNA molecule characterized by substantial structural heterogeneity [16]. The circular structure of ecDNA confers markedly higher chromatin accessibility [17]. This open conformation enhances the recruitment of transcription factors and RNA polymerases, which dramatically enhances oncogene expression. Concurrently, it may preserve stability by avoiding DNA end exposure, thereby preventing exonuclease-mediated degradation [18, 19]. Notably, ecDNA often harbors enhancers and super-enhancers that can engage in non-linear spatial interactions with oncogenes, which is known as enhancer hijacking [5, 12] **(**Fig. 1A**)**. This configuration bypasses the topologically associating domain constraints of chromosomal DNA. As a result, oncogenes on ecDNA can be robustly activated by distal or ectopic enhancers, leading to markedly elevated transcriptional output.

### EcDNA has unique chromatin and epigenetic landscape

Although ecDNA is also packaged as a chromatin consisting of nucleosomes, it exhibits key differences in its structure compared with that of chromosomal DNA. Unlike chromosomal DNA, which is tightly compacted into higher-order structures that limit transcriptional accessibility, ecDNA lacks such compaction [17] **(**Fig. 1B**)**. This open architecture promotes widespread chromatin accessibility, thereby driving oncogene transcription.

Notably, histone modifications associated with active regulatory regions, such as histone acetylation and enrichment of marks like H3K4me1/3 and H3K27ac, exhibit distinct patterns on ecDNA compared to chromosomal DNA [17]. In addition, ecDNA harbors fewer repressive histone marks (H3K9me3 and H3K27me3) **(**Fig. 1B**)**. These epigenetic signatures are often linked to enhancer activity and heightened transcriptional potential [17]. Promoter regions on ecDNA also tend to be hypomethylated, which facilitates increased gene expression **(**Fig. 1B**)**. The integration of CRISPR-CATCH with nanopore sequencing has enabled single-molecule methylation profiling of ecDNA [20]. In glioblastoma models, this approach revealed that the EGFR promoter on ecDNA exhibits marked hypomethylation, which significantly enhances EGFR expression and contributes to tumor progression [20].

### EcDNA hubs boost transcription

In some cancers, ecDNA is not randomly distributed in the nucleus. Instead, it tends to form locally aggregated “ecDNA hubs”, which are thought to be associated with amplifying oncogene expression [21] **(**Fig. 1C**)**. Within ecDNA hubs, distinct ecDNA species spatially converge to share regulatory elements. For instance, in the gastric cancer SNU16 cell, ecDNAs carrying MYC and FGFR2 co-localize to form a transcriptionally active hub, where enhancers on FGFR2-ecDNA can activate the MYC promoter in trans, thereby upregulating MYC expression [22]. These ecDNA hubs are dynamic assemblies, and their size correlates positively with RNA polymerase II occupancy, suggesting that larger hubs mediate higher levels of oncogene transcription [23]. Beyond interactions between ecDNA elements, ecDNA hubs can also engage in regulatory crosstalk with chromosomal DNA, activating gene expression from chromosomally encoded loci and expanding the transcriptional regulatory landscape of cancer cells [24]. The formation and maintenance of ecDNA hubs is thought to be mediated by bromodomain and extraterminal domain (BET) protein BRD4, which promote enhancer-promoter interactions [22].

## Biogenesis, replication and transcription of EcDNA

### Biogenesis

During tumor progression, cancer cells undergo rapid proliferation and are exposed to considerable external stress. These conditions increase the frequency of chromosome segregation errors during mitosis, which in turn leads to sustained karyotypic reorganization-a hallmark of chromosomal instability (CIN) [25, 26]. This instability has led to the existence of a number of ecDNA beyond the regular chromosomal genome, which are highly amplified and transcriptionally active. Several currently recognized CIN conditions linked to ecDNA generation are elaborated upon in the following section.

#### DNA double-strand breaks

**Fig. 2:**
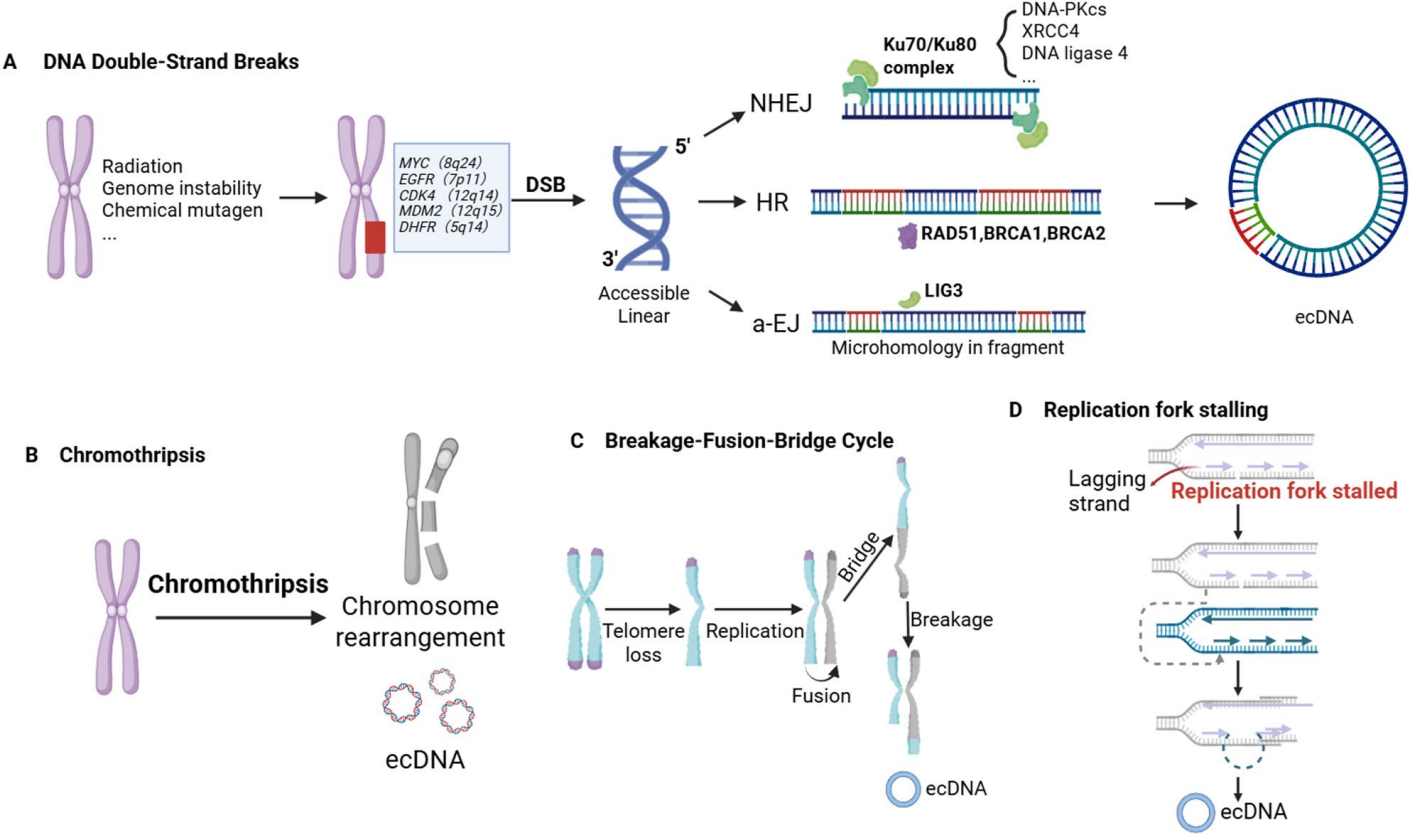
An illustration of the formation mechanism of ecDNA. **A** When DNA double-strand break occurs, the resulting DNA fragment can religate to form ecDNA. DNA repair pathways playing crucial roles in the circularization of the fragmented DNA. **B** During chromothripsis, more DNA fragments are generated. ecDNA may arise from DNA fragments that are unable to reintegrate into the chromosome. **C** In the Breakage-Fusion-Bridge Cycle, loss of chromosomal telomeres leads to the production of rearranged chromosomes and ecDNA. **D** When DNA replication is stalled, template strand replacement may occur, leading to mismatched base pairing and the extrusion of looped DNA structures that contribute to the emergence of ecDNA. Figure created using BioRender (biorender.com)

Highly amplified genes display two structures: intrachromosomal homogeneously stained regions (HSRs) and ecDNA [27]. Increasing experimental evidence indicates that ecDNA may arise from DNA double-strand breaks (DSBs), whose error-prone repair promotes the circularization of excised chromosomal fragments. The two major DNA repair pathways for DSBs are NHEJ and homologous recombination (HR). In addition, DSBs end joining can also occur through a mechanism independent of the classical NHEJ pathway, known as alternative end joining (a-EJ). This repair mode includes MMEJ in yeast and theta-mediated end joining (TMEJ) in humans [28]. CRISPR-engineered DSBs models have directly induced de novo ecDNA with microhomology signatures, consistent with NHEJ/MMEJ-mediated ligation [13] (Fig. 2A).

While NHEJ and MMEJ are known to contribute to ecDNA generation, the regulatory mechanisms coordinating their involvement remain unclear. Studies have demonstrated that DNA-PKcs inhibitor (DNA-PKi; involved in NHEJ) and ATM inhibitor (ATMi; involved in MMEJ) have opposing effects on ecDNA formation [13]. DNA-PKi promotes the aggregation of DSBs, thereby promoting ecDNA formation, while ATMi limits the mobility and aggregation of DSBs [13]. In addition, Cai et al. found that inhibition of HR reduced ecDNA amplification and reversed methotrexate (MTX) resistance in drug-resistant cells, suggesting that HR may participate in ecDNA formation. However, the precise underlying mechanism remains unclear [29]. In another study, the BRCA1-A and LIG4 complexes were found to participate in ecDNA production [30]. The BRCA1-A complex facilitates LIG4-mediated circularization by protecting DNA ends from excessive excision [30]. LIG4, a core component of the NHEJ pathway, and the BRCA1-A complex, which acts upstream in the DNA damage response and critically regulates HR, collectively indicate that the biogenesis of ecDNA is not governed by a single DNA repair pathway [31–33]. In summary, current literature generally posits that DSBs are the initiating events in ecDNA formation, with subsequent DNA repair pathways playing crucial roles in the circularization of the fragmented DNA.

#### Chromothripsis

In addition to the above-mentioned DSBs, destabilization of the cancer genomes can lead to the acquisition of chromosomal abnormalities, such as chromothripsis. Chromothripsis is the best-known origin of ecDNA, in which chromosomes break into small pieces as a result of catastrophic DNA damage and undergo extensive DNA rearrangements through replication, a process that leads to complex localized genomic rearrangements in up to one-third of cancer cells [34, 35]. Approximately 36% ecDNA amplicons were found to show signatures of chromothripsis [9]. Chromothripsis generates multiple DNA breaks, providing a sufficient substrate for ecDNA cyclisation and recombination. In addition, chromothripsis is not only a mechanism for ecDNA formation, but may also lead to further evolution and rearrangement of ecDNA, thereby enhancing drug resistance in tumors [36]. It has been shown that ecDNA itself can undergo successive chromothripsis, resulting in the production of a more therapy-resistant, rearranged ecDNA [36] (Fig. 2B). Engel et al. reported that the Fanconi anemia (FA) pathway is capable of inducing chromothripsis, generating complex genomic rearrangements and ecDNA to obtain acquired resistance to anticancer therapy [37].

#### Breakage-fusion-bridge cycle

The breakage-fusion-bridge (BFB) cycle is a mechanism of chromosome instability potentially leading to gene amplification and ecDNA formation [38]. The process begins with the breakage and deletion of telomeres, which results in the fold-back inversion of sister chromatids. This inversion generates a dicentric chromosome that subsequently forms a fused bridge [39]. As cell division proceeds, the BFB cycle persists until the affected chromosome gains a new telomere, thereby halting the cycle [40]. The BFB cycle can generate new ecDNA while promoting genomic instability and even chromothripsis [41] (Fig. 2C).

#### Replication fork stalling and replication template abnormalities

During rapid tumor proliferation in vivo, cancer cells are experiencing adverse environmental factors such as hypoxia, highly acidic tumor microenvironment, and nutrient deprivation [42, 43]. These stressors collectively impose substantial replication stress, a hallmark of which is the stalling or collapse of replication forks [44]. Fork stalling can disrupt normal lagging strand synthesis, triggering aberrant template switching, whereby DNA polymerase disengages and transiently anneals to nearby single-stranded DNA regions to continue DNA synthesis [45]. The lagging strand intrusion and recombination might occur at any time during this process, which may occur repeatedly until the lagging strand eventually returns and serves as the template strand. Such events can result in structural abnormalities, including tandem duplications [46, 47]. During this process, incomplete complementarity between the newly synthesized DNA and the original template may lead to the formation of single-stranded loops. These loop structures are hypothesized to serve as precursors to ecDNA (Fig. 2D) [48].

### Replication

Although ecDNA lacks the centromeres and telomeres of chromosomes, it still replicates during the S phase, and utilizes the conventional replication machinery [9, 17]. Compared with chromosomes, the origins of replication in ecDNA are more randomly distributed and tend to occur in open chromatin regions and areas rich in enhancers and driver genes [17]. The high transcriptional activity of ecDNA often overlaps with replication, leading to replication-transcription conflicts (TRCs), a form of molecular interference arising when transcription and replication machineries converge on the same DNA template. This overlapping region elicits topological strain, leading to stalling of replication forks, induction of replication stress, and the eventual occurrence of DSBs [49]. These damages are sensed by the DNA damage response pathways, including the ATM/ATR signaling pathways, and are primarily repaired by alternative non-homologous end-joining (alt-NHEJ), which is crucial for maintaining the circular structure and copy number stability of ecDNA [49].

### Transcription

In terms of transcription, ecDNA exhibits an open chromatin state and is rich in active histone modifications (such as H3K27ac and H3K4me3), which facilitates the binding of transcription factors [17]. Multiple ecDNA molecules often aggregate to form “ecDNA hubs”, and frequent enhancer-promoter interactions greatly enhance the expression of oncogenes. These aggregates are mainly localized in nuclear regions, such as nuclear speckles or condensed areas, recruiting RNA polymerase II and coactivators [22, 23]. Importantly, the unequal distribution of ecDNA during cell division leads to heterogeneity in oncogene copy numbers among daughter cells, thereby driving variability in transcriptional output and promoting tumor heterogeneity and adaptive evolution [23].

## Function of EcDNA in human cancer

### EcDNA is associated with cancer initiation

EcDNA has emerged as a hallmark of oncogenic genome architecture, present predominantly in cancer cells and rarely detected in healthy human cells [50]. This cancer-specific distribution makes ecDNA an attractive target for precision diagnostics and prognostics. A pan-cancer analysis of over 14,000 patients from the 100,000 Genomes Project revealed that ecDNA is detectable in approximately 17.1% of cases [11], with markedly higher prevalence in tumors such as colorectal cancer (CRC), glioblastoma multiforme, and esophageal adenocarcinoma (EAC) [51–53]. Notably, ecDNA appears to emerge early during malignant transformation. In patients with Barrett’s esophagus who subsequently developed EAC, ecDNA was retrospectively identified in premalignant biopsies at a significantly higher frequency than in those who did not progress to cancer [52]. Approximately 33% of patients later diagnosed with EAC had ecDNA-positive biopsies prior to diagnosis, implicating ecDNA as a potential early indicator of neoplastic evolution [52].

Since ecDNA is closely involved in tumorigenesis, whether it can serve as a potential indicator for predicting tumor occurrence has become a focus of clinical research. Notably, liquid biopsy has been increasingly widely used in the early diagnosis of tumors in recent years, and the detection of ecDNA in body fluids is just a key exploration direction under this technical framework. While small eccDNAs or fragmented DNA can be detected in circulating body fluids [54–56], cancer-associated ecDNAs are typically hundreds of kilobases to megabases in size. Therefore, ecDNAs are thought to exist at very low abundance in body fluids, and studies utilizing ecDNA detection in liquid biopsy for tumor prediction are relatively rare. Interestingly, in urothelial carcinoma, urinary ecDNA shows strong genomic concordance with tumor samples, albeit with lower detection sensitivity [57]. Importantly, specificity was retained across clinical subgroups and tumor purities, underscoring ecDNA’s diagnostic robustness across heterogeneous disease contexts [57]. However, current research on ecDNA-based liquid biopsy remains limited, and further evidence is needed to establish its clinical utility.

### EcDNA is associated with cancer progression and prognosis

EcDNA frequency increases during tumor evolution. It is more prevalent in untreated metastatic and pretreated tumors than in newly diagnosed primary cancers [58]. This enrichment correlates with tumor aggressiveness, genetic instability, and poor clinical outcomes [58]. Oncogene amplification via ecDNA accelerates tumor evolution and fosters drug resistance, and thereby significantly shortens patient survival. In addition, these mechanisms work in concert to amplify the driving effect of amplified genes [17, 22].

For instance, in small-cell lung cancer (SCLC), MYC-driven ecDNA amplification is a key driver of intratumoral heterogeneity and phenotypic plasticity between neuroendocrine and non-neuroendocrine states [5]. SCLC patients harboring such complex ecDNA configurations have significantly shorter survival durations [5].

Mechanistically, ecDNA often carries complete oncogenes (such as MYC, EGFR, FGFR2, etc.) and significantly enhances their expression through high-level amplification, thereby driving excessive cell proliferation. This dosage effect is usually far greater than that of chromosomal amplification [8, 59]. The unique structural features of ecDNA (see details in the section of the structural and functional characteristics of ecDNA), including its higher chromatin accessibility, reduced enrichment of repressive histone modifications (H3K9me3 and H3K27me3) and DNA methylation, together with increased levels of active histone marks (H3K4me1/3 and H3K27ac), collectively contribute to a highly permissive transcriptional environment [17, 20]. Moreover, enhancer hijacking events and the formation of ecDNA hubs further amplify oncogene copy number and transcriptional output, and thereby promote tumor progression [6, 12, 22]. Furthermore, owing to its asymmetric segregation during somatic cell mitosis and capacity for high-level oncogene amplification, ecDNA promotes intratumoral heterogeneity, which is a critical determinant of therapeutic failure and disease progression [8].

### EcDNA is associated with therapeutic resistance in cancer

Emerging evidence has revealed that ecDNA plays a pivotal role in driving resistance to cancer therapies, contributing to both the molecular evolution of tumors and the failure of clinical treatments [60].

One of the most studied examples involves MTX resistance in CRC. MTX competitively inhibits the activity of dihydrofolate reductase (DHFR), blocking the reduction of dihydrofolate to tetrahydrofolate, and thus inhibiting DNA synthesis [61]. The earliest reports of MTX resistance identified DHFR gene amplification on ecDNA as the primary mechanism of resistance [62]. Recent genomic studies confirm that ecDNA harboring DHFR and other genes such as MSH3 and FAM151B drives robust MTX resistance in CRC [63]. Similarly, amplification of FGFR2 on ecDNA in gastric cancer confers resistance to FGFR inhibitors such as infigratinib [64], while ecDNA-mediated BRAF amplification has been implicated in resistance to BRAF and MEK inhibitors in melanoma [65]. In prostate cancer (PCa), ecDNA has been shown to mediate resistance to androgen signaling inhibitors. Metastatic castration-resistant prostate cancer (mCRPC) often exhibits androgen receptor (AR) gene amplification on ecDNA, which promotes escape from androgen blockade therapies [66, 67]. Interestingly, the loss of ecDNA is not always beneficial. Instead, it may serve as an inducer of the drug resistance mechanism in tumor cells. In glioblastoma, tumor cells expressing ecDNA-amplified EGFRvIII (constitutively active EGFR mutant) are initially sensitive to EGFR tyrosine kinase inhibitors (TKIs). However, resistance emerges via selective loss of ecDNA containing EGFRvIII, and notably, these ecDNAs reappear following drug withdrawal, indicating a reversible resistance mechanism [53] (Table 1).

**Table 1:** EcDNA mediates cancer therapeutic resistance

Key genes on ecDNA	Cancer type	Corresponding role	Ref.
DHFR	CRC	DHFR amplification on DMs drives MTX resistance in tumor cells.	[68]
DHFR, MSH3, FAM151B, ZFYVE16	CRC	EcDNA drives robust amplification of DHFR and other genes, leading to MTX resistance.	[63]
FGFR2	Gastric cancer	FGFR2-amplified ecDNA promotes the acquisition of resistance of tumor cells to the pan-FGFR tyrosine kinase inhibitor infigratinib.	[64]
RAB3B	Hypopharyngeal squamous cell carcinoma	RAB3B exists in linear chromosomal and circular ecDNA and promotes cisplatin resistance.	[69]
BRAF	Melanoma	Resistance to BRAF and MEK inhibitors is conferred by focal genomic amplifications, specifically via DMs and HSRs.	[65]
MYC, MYCN, and MYCL	SCLC	EcDNA carrying MYC paralogs induce acquired cross-resistance.	[10]
MET	ROS1 + NSCLC	EcDNA-mediated MET amplification confers resistance to entrectinib therapy.	[70]
MYC	Pancreatic ductal adenocarcinoma	EcDNA-driven MYC overexpression confers resistance to the Porcupine inhibitor C59 (blocks endogenous WNT ligand production).	[71]
AR	PCa	mCRPC with ecDNA-amplified AR exhibits increased resistance to androgen signaling inhibitors	[66, 67]
CCND1	Urothelial carcinoma	EcDNA-driven CCND1 mediates adaptive resistance to cisplatin chemotherapy	[72]
EGFRvIII	Glioblastoma	Resistance of tumor cells to TKIs is associated with a reduction of ecDNA containing EGFRvIII.	[53]

*ecDNA* extrachromosomal DNA, *CRC* colorectal cancer, *DMs* double minutes, *HSRs* homogeneously stained regions, *SCLC* small-cell lung cancer, *PCa* prostate cancer, *mCRPC* metastatic castration-resistant prostate cancer, *TKIs* tyrosine kinase inhibitors.

ecDNA not only mediates therapy resistance through oncogene amplification but also influences immune evasion. Several studies have shown that ecDNA + tumors exhibit reduced MHC class I and II gene expression, impairing antigen presentation and T cell recognition [57, 73]. Furthermore, ecDNA often carries immune-regulatory genes involved in the negative regulation of cytotoxicity and lymphocyte activation, contributing to a broadly immunosuppressive tumor microenvironment [11]. In ecDNA-rich tumor environments, global transcriptional suppression of immune functions has also been reported, including cancer antigen presentation, the trafficking of T cells, and the infiltration and recognition of tumor cells by cytotoxic T cells within the cancer immune cycle [74, 75]. In gastric and nasopharyngeal carcinomas, ecDNA amplification correlates with poor response to PD-1 blockade, independent of PD-L1 expression or tumor mutational burden, underscoring its value as a biomarker for immunotherapy stratification [76]. Although some immune checkpoint ligands, such as PD-L1, can occasionally be amplified on ecDNA—as seen in rare HPV-associated oropharyngeal cancers [77], bulk analyses show that ecDNA + tumors generally downregulate checkpoint gene expression [73]. Notably, most insights into the immunomodulatory functions of ecDNA remain correlative and genomics-based. There is a pressing need for mechanistic and functional studies to dissect causal pathways and validate therapeutic vulnerabilities. Understanding ecDNA’s dual role in resistance and immune escape may unlock new strategies for overcoming therapeutic failure and improving cancer patient outcomes.

## Targeting strategies against EcDNA in cancer

EcDNA is a potent oncogenic driver found predominantly in cancer cells, rendering it an attractive and tumor-specific therapeutic target. Unlike chromosomal DNA, ecDNA facilitates high-level oncogene amplification and contributes to treatment resistance and tumor evolution [78]. Therapeutic strategies targeting ecDNA aim to interfere with its key biological processes, including biogenesis, replication and transcription, transcription–replication conflict, segregation, clustering, and clearance. These approaches aim to eliminate ecDNA-positive tumor cells or neutralize their oncogenic potential.

### Inhibiting EcDNA biogenesis

While ecDNA-driven tumors harbor pre-formed ecDNA populations, inhibiting biogenesis remains a valid clinical strategy for three interrelated reasons. First, ecDNA pools are not static but undergo continuous turnover. Even in advanced tumors, ongoing DNA damage drives de novo ecDNA formation, which fuels genetic heterogeneity and resistance [36, 49]. Pharmacological inhibition of DNA repair enzymes disrupts this process, preventing the emergence of new ecDNA variants that could confer adaptive resistance during treatment [30, 79]. For instance, DNA-PKcs inhibition reduces ecDNA size and complex rearrangements in melanoma models, delaying resistance to MAPK inhibitors [79]. Second, ecDNA replication and segregation are inherently error-prone, leading to frequent loss of ecDNA copies in daughter cells. By blocking biogenesis, the replenishment of ecDNA is disrupted, shifting the balance toward net ecDNA depletion. Preclinical studies show that combined inhibition of DNA repair and ecDNA circularization reduces ecDNA copy numbers in established tumor models, sensitizing cells to chemotherapy [30]. Third, biogenesis pathways overlap with those driving tumor evolution. Mechanisms like chromothripsis and the BFB cycle, which generate ecDNA, also promote chromosomal instability underlying metastasis and drug resistance [37, 41]. Targeting biogenesis exerts dual effects: inhibiting ecDNA expansion while disrupting broader genomic rearrangements [13, 37]. Thus, inhibiting ecDNA biogenesis is not limited to preventing initial formation but also destabilizes pre-existing ecDNA by interrupting their regenerative capacity and evolutionary adaptation, making it a viable strategy for both primary and drug-resistant tumors.

The circular structure of ecDNA lacks free DNA ends, making it more resistant to exonuclease-mediated degradation compared to linear DNA [80]. A major therapeutic challenge lies in selectively disrupting ecDNA circularization or destabilizing already-formed circular structures. During the formation of ecDNA, the BRCA1-A complex protects DNA ends from excessive resection and directs them to LIG4 for end-to-end ligation [30]. Pharmacological inhibition of LIG4 has been proposed as a means to impair the circularization process, thereby sensitizing ecDNA intermediates to nucleolytic degradation [30]. Recent studies have shown that the loss of alt-NHEJ impairs the repair of DNA DSBs in a cell cycle-dependent manner, thereby interfering with the circularization of ecDNA [81]. This disruption may lead to the reintegration of ecDNA into chromosomal DNA or its elimination from the cell [81]. Targeting the molecular machinery that enables ecDNA circularization may offer a tumor-selective therapeutic window, particularly in cancers with a high ecDNA burden.

### Blocking EcDNA replication and transcription

EcDNA depends on robust replication and transcription programs for its maintenance and oncogenic activity. Hydroxyurea, a ribonucleotide reductase (RNR) inhibitor, competitively blocks the synthesis of dNTPs by inhibiting class I RNR activity, thereby depleting the nucleotide pool essential for DNA replication [82]. This reduction in precursor availability selectively disrupts the replication of ecDNA, which requires continuous synthesis to maintain oncogene copy number. Both in vitro and in vivo studies have demonstrated that hydroxyurea treatment leads to the depletion of ecDNA harboring oncogenes or resistance genes, ultimately resulting in tumor cell death [83, 84]. Epigenetically, ecDNA loci are enriched for active chromatin modifications such as histone H3K27ac and H3K4me3, both of which facilitate elevated transcription of ecDNA-encoded oncogenes [85]. Inhibiting these histone modifications or their associated readers and writers may represent a complementary strategy to reduce ecDNA transcriptional output.

### Enhancing transcription–replication conflict

Additionally, the structural and transcriptional properties of ecDNA exacerbate replication stress through TRCs. The circular configuration and enhancer-rich landscape of ecDNA lead to increased transcriptional activity, which in turn amplifies replication pressure, single-stranded DNA exposure, and the accumulation of DNA damage [64]. In this context, inhibition of CHK1, a key mediator of the DNA damage response, has been shown to intensify replication stress and induce selective lethality in ecDNA-containing tumor cells [64]. This mechanism underpins an ongoing phase I/II clinical trial (NCT05827614) focusing on patients with locally advanced or metastatic solid tumors characterized by oncogene amplification. Specifically, the trial assesses the CHK1 inhibitor BBI-355 both as monotherapy or in combination with erlotinib or futibatinib.

### Targeting EcDNA segregation and clustering

Although ecDNA lacks centromeres, it is efficiently transmitted during mitosis through a tethering mechanism involving telomeric and subtelomeric chromosomal regions [86]. This spindle-dependent segregation ensures the faithful inheritance of ecDNA across cell generations, supporting the maintenance of oncogene amplification. While the precise function of ecDNA-telomere tethering remains incompletely understood, emerging evidence suggests a potential role for telomeric repeat-containing RNA and telomere-associated transcription in stabilizing ecDNA-chromosome interactions [87]. In MYC-driven tumors, inhibition of BRD4, depletion of nascent lncRNA PVT1 encoded on MYC-containing ecDNA, or suppression of mitotic transcription has been shown to disrupt ecDNA segregation fidelity by targeting mitotic tethering points. This leads to the mislocalization of ecDNA, followed by its reintegration into the genome as HSRs, which are often less transcriptionally active [86]. Furthermore, in cancer cells harboring multiple ecDNA species, distinct ecDNA circles often exhibit cooperative inheritance patterns, preferentially co-segregating into the same daughter cells [88]. This phenomenon appears to be driven by spatial proximity and coordinated transcriptional activity. Inhibiting transcription initiation with triptolide can prevent this co-segregation behavior, further underscoring the link between ecDNA activity and mitotic behavior [88]. Beyond segregation, ecDNA molecules often self-organize into nuclear clusters known as ecDNA hubs. Disruption of ecDNA hubs formation impairs the transcriptional activity of ecDNA-based oncogenes. In CRC cell lines harboring MYC amplification, ecDNA hubs are orchestrated by BRD4. Pharmacological inhibition of BRD4 with the BET inhibitor JQ1 disrupts ecDNA hub integrity, thereby suppressing oncogene transcription [22]. While BRD4 is a key organizer, the full complement of proteins involved in ecDNA clustering remains to be elucidated. Identifying additional hub-associated factors may uncover new therapeutic targets capable of attenuating oncogenic activity of ecDNA.

### Promoting EcDNA clearance

Recent studies reveal that endogenous nucleases can be pharmacologically leveraged to promote ecDNA clearance. In hepatocellular carcinoma, vitamin D (VD) stabilizes the nuclease DNase1L3 through its binding partner GC, enabling targeted degradation of ecDNA located around nuclear lipid droplets [89]. Two mRNA-based therapeutics, DNase1L3 mRNA and GC-DNase mRNA, showed significant antitumor efficacy in patient-derived xenograft models. These results indicated that pharmacological ecDNA clearance may represent a viable therapeutic modality [89].

Importantly, inhibition of ecDNA-encoded oncogenes or drug-resistance genes can promote the elimination of ecDNA via micronuclei formation. This phenomenon leads to a reduction in ecDNA copy number and suppresses sustained gene amplification [90]. Low-dose hydroxyurea mediates the nuclear aggregation of DMs, which consequently generates micronuclei [91]. Furthermore, fractionated radiation therapy has been shown to enhance micronuclear entrapment of ecDNA, further impeding its oncogenic amplification cycle [92]. Notably, CRISPR/Cas9 can be used to induce DSBs in ecDNA within CRC cells, promoting micronuclei formation and the subsequent clearance of ecDNA [91]. Such an approach can disrupt ecDNA-dependent oncogene amplification and may consequently suppress tumor cell growth. However, this strategy has so far been demonstrated only in vitro and lacks validation in in vivo models.

## Conclusions and perspectives

EcDNA is widely present across various human cancers and has been established as a critical driver of rapid tumor evolution, elevated oncogene expression, enhanced intratumoral heterogeneity, and therapeutic resistance [9, 93]. Its distinct circular structure and centromere-independent replication enable asymmetric segregation during mitosis, thereby conferring exceptional adaptability and survival advantages to cancer cells [94].

Although recent studies leveraging high-throughput sequencing technologies have significantly advanced our understanding of the structure and function of ecDNA, the mechanisms underlying its formation and maintenance remain poorly elucidated. How is ecDNA replicated and maintained, and how does it interact with chromosomes during cell division? Future research should address how DNA repair pathways such as HR, NHEJ, and a-EJ contribute to ecDNA biogenesis. At the transcriptional level, ecDNA reshapes oncogene networks through enhancer-promoter rewiring and the formation of ecDNA hubs, thereby reprogramming the transcriptional landscape and promoting malignant adaptation [22]. A deeper understanding of these transcriptional and epigenetic consequences will facilitate the identification of vulnerabilities that can be therapeutically exploited.

EcDNA holds significant promise in clinical oncology, particularly for facilitating early tumor diagnosis and guiding treatment decisions [57, 76]. Its tumor-specific occurrence makes it an attractive and specific biomarker for diagnostic applications. However, the prevalence of ecDNA across all tumor types remains relatively low [11]. Can ecDNA content and structural heterogeneity serve as robust biomarkers for early diagnosis and risk stratification? Future efforts should focus on standardizing ecDNA detection and quantification across sample types and analytical platforms, and on enhancing the sensitivity and specificity of liquid biopsy-based detection methods.

**Fig. 3:**
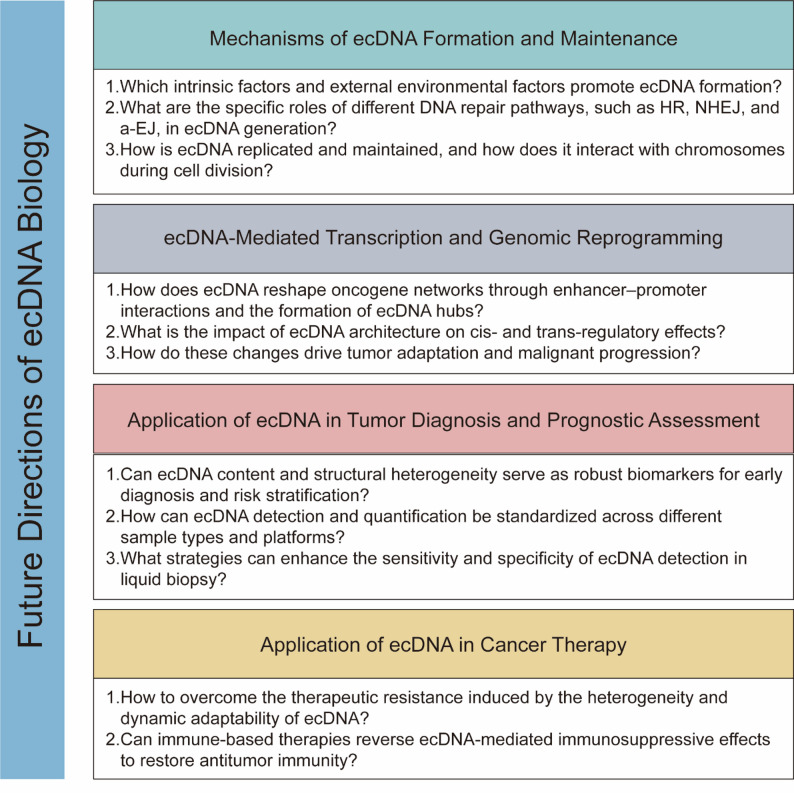
Future directions of ecDNA Biology

Current strategies predominantly focus on disrupting the processes involved in ecDNA biogenesis and maintenance. These novel therapeutic approaches aim to exploit the unique biological properties of ecDNA. However, major challenges remain in overcoming the therapeutic resistance driven by the intrinsic heterogeneity and dynamic adaptability of ecDNA. Moreover, accumulating evidence has indicated that ecDNA contributes to tumor immune evasion. This highlights the potential value of exploring immune-based interventions, which may reverse ecDNA-mediated immunosuppressive effects and restore antitumor immunity to open new therapeutic avenues [74, 75] (Fig. 3).

## Data Availability

No datasets were generated or analysed during the current study.

## Abbreviations

EcDNA: Extrachromosomal DNA
EccDNA: Extrachromosomal circular DNA
DMs: Double minutes
NHEJ: Nonhomologous end joining
MMEJ: Microhomology-mediated end joining
BET: Bromodomain and extraterminal domain
CIN: Chromosomal instability
HSRs: Homogeneously stained regions
DSBs: Double-strand breaks
HR: Homologous recombination
TMEJ: Theta-mediated end joining
MTX: Methotrexate
FA: Fanconi anemia
BFB: Breakage-fusion-bridge
TRCs: Replication-transcription conflicts
CRC: Colorectal cancer
EAC: Esophageal adenocarcinoma
SCLC: Small-cell lung cancer
DHFR: Dihydrofolate reductase
PCa: Prostate cancer
MCRPC: Metastatic castration-resistant prostate cancer
AR: Androgen receptor
TKIs: Tyrosine kinase inhibitors
RNR: Ribonucleotide reductase
